# Cartilage oligomeric matrix protein-induced complement activation in systemic sclerosis

**DOI:** 10.1186/ar4410

**Published:** 2013-12-13

**Authors:** Kaisa E Otteby, Emelie Holmquist, Tore Saxne, Dick Heinegård, Roger Hesselstrand, Anna M Blom

**Affiliations:** 1 Section of Medical Protein Chemistry, Department of Laboratory Medicine, Lund University, Skåne University Hospital, S-20502 Malmö, Sweden; 2 Department of Clinical Sciences, Section of Rheumatology, Lund University, Skåne University Hospital, S-22185 Lund, Sweden

## Abstract

**Introduction:**

Complexes between cartilage oligomeric matrix protein (COMP) and the complement activation product C3b have been found in the circulation of patients with rheumatoid arthritis and systemic lupus erythematosus. In systemic sclerosis (SSc) COMP expression in the skin is upregulated both in lesional and non-lesional skin, which is also reflected in an increased amount of circulating COMP. We investigated the presence of COMP-C3b complexes in serum and skin biopsies of patients with SSc.

**Methods:**

The presence of COMP and COMP-C3b complexes in the serum of 80 patients with limited cutaneous SSc (lcSSc, n = 40) and diffuse cutaneous SSc (dcSSc, n = 40) and 97 healthy controls was measured by ELISA and correlated to different clinical parameters. Samples were collected both at baseline and after three to five years to assess longitudinal changes in COMP-C3b complex levels. Furthermore, skin biopsies from seven patients with dcSSc and three healthy controls were analyzed for expression of COMP and deposition of C3b and IgG.

**Results:**

Serum levels of COMP-C3b were found to be elevated in both dcSSc and lcSSc compared to healthy controls and decreased at the second measurement in patients on immunosuppressive therapy. No co-localization of COMP and C3b was found in the skin biopsies, indicating that the COMP-C3b complexes are formed upon release of COMP into the circulation.

**Conclusion:**

COMP-C3b complexes are found in the serum of patients with SSc. The lack of co-localization between COMP and C3b in the skin suggests that COMP does not drive complement activation in the skin in SSc.

## Introduction

Systemic sclerosis (SSc) is a multi-organ disease characterized by fibrosis of the skin and internal organs as well as vasculopathy [1]. SSc is generally divided into two subsets based on the extent of skin fibrosis and organ involvement; diffuse cutaneous SSc (dsSSc) or limited cutaneous SSc (lcSSc). Fibrosis in SSc is associated with extensive deposition of extracellular matrix components, such as collagen type I, III [2] and V [3] in the affected tissue. Several studies have also reported the presence of cartilage oligomeric matrix protein (COMP) in both lesional and non-lesional skin biopsies [4-6] as well as expression in cultured dermal fibroblasts from SSc patients [4,7]. Others have also found COMP in healthy human skin where it was suggested to regulate the structure of the collagen I network [8]. COMP, also known as Thrombospondin-5, is a pentameric protein involved in the assembly and stabilization of collagen networks in extracellular matrices [9,10]. COMP was originally purified as a component of cartilage [11], but is also expressed in tendon [12]. COMP is found at elevated levels in serum during SSc, most likely as a result of release from the affected tissues, and serum COMP has been shown to correlate with the modified Rodnan skin score (mRSS) indicating that the amount of COMP released into the circulation is dependent on disease activity and/or severity related to fibrosis [13]. Serum COMP has been found to be higher in patients with dcSSc than in patients with lcSSc and may further be elevated by SSc-related arthritis [5,14]. Serum COMP levels in early disease have in addition been shown to predict mortality in SSc, possibly explained by the more frequent and severe internal organ involvement in patients with dcSSc [15]. Although COMP is also expressed in vascular smooth muscle cells [16], serum COMP levels in SSc have mostly reflected features of fibrosis and not vascular complications.

Activation of the immune system is a critical feature of SSc. Infiltrates of activated T-cells and macrophages are found in skin lesions early in disease [17,18]. Inflammation is often less pronounced in SSc than in other rheumatic diseases but increased serum levels of pro-inflammatory cytokines, such as tumor necrosis factor, interferon-γ and interleukin-6 can be found in SSc patients and are influenced by both disease duration and the autoantibody profile [19,20]. Autoantibodies can be found in the majority of patients, most commonly against centromere, topoisomerase I, U3/U1-RNP and RNA polymerase III [21], although the contribution of such antibodies to disease pathology is still unknown. Activation of the complement system is also likely to occur, as complement activation products C3d, Ba [22] and C4a [23] can be found in the circulation of patients during active disease. Furthermore, C5b-9 can be found in skin lesions in both early and advanced SSc [24]. A decreased expression of the complement inhibitors decay accelerating factor and membrane cofactor protein in the vascular endothelium has also been observed in SSc, which might contribute to vascular damage and further to fibrosis [25].

We showed previously that COMP can activate the complement system, which occurs exclusively through the alternative pathway [26]. Furthermore, as an indication of *in vivo* complement activation by COMP, complexes between COMP and the complement activation product C3b can be found in the serum of patients with SSc [27]. Whether these complexes are formed in the skin lesions or in serum after COMP-release is, however, still unclear. By studying extracts of skin biopsies from SSc patients, it has been demonstrated that COMP in the skin comprises an approximately 56 kDa region of the C-terminus [13]. Interestingly, the C-terminus activates complement [26] and it also stimulates autoantibody production in patients with rheumatoid arthritis [28]. Therefore we hypothesized that COMP induces complement activation in the skin at the site of COMP-expression.

In this study we set out to verify the presence of COMP-C3b complexes in the serum of SSc patients using a larger patient cohort and to study the relation of COMP-C3b to different clinical parameters as well as to examine presence of complexes longitudinally. We have further investigated the presence and co-localization of COMP and deposited complement components in the skin of SSc patients.

## Methods

### Patients

Serum was collected from 80 patients with SSc who all fulfilled the American College of Rheumatology (ACR) criteria for SSc [29]. The disease was classified as dcSSc (n = 40) or lcSSc (n = 40) based on the extent of skin involvement [30]. Samples were collected within 3 years of disease onset, which was defined as the first non-Raynaud’s manifestation. A second sample was collected from each patient 3 to 5 years after the first sampling. Of the 80 patients, 20 were included in a previous cross-sectional study on COMP-C3b in SSc [27].

Serum was furthermore collected from 97 healthy volunteers with no history of rheumatologic disease, from Lund and Malmö. All serum samples were retrieved at a standardized fashion (non-fasting) and were stored at −80°C after centrifugation. Further characteristics of the patients and controls are described in Table 1.

**Table 1:** Description of patients and controls

	**lcSSc baseline**	**lcSSc second sample**	**dcSSc baseline**	**dcSSc second sample**	**Controls**
Number	40	40	40	40	97
Age, years median (range)	47.5 (16 to 77)	50 (21 to 81)	51.5 (22 to 77)	56 (26 to 80)	45 (23 to 74)
Gender, female:male	33:7	33:7	28:12	28:12	67:30
COMP, U/l median (range)	9.3 (4.2 to 27.9)	9.7 (3.9 to 15.8)	16.0 (6.2 to 37.2)	10.9 (5.0 to 30.0)	7.0 (3.2 to 12.1)
Disease duration, years median (range)	1.4 (0.2 to 3.0)	5.0 (3.2 to 7.1)	1.0 (0.3 to 3.0)	4.6 (3.3 to 7.5)	Not applicable
COMP-C3b, AU median (range)	2.5 (0.4 to 6.4)	1.9 (0.8 to 6.1)	2.3 (0.2 to 3.8)	1.7 (0.7 to 4.0)	0.6 (0 to 4.4)
mRSS median (range)	5.0 (2.0 to 19.0)	3.0 (0 to 15.0)	22.5 (3.0 to 43.0)	12.5 (0 to 33.0)	Not applicable
CRP, mg/l median (range)	5.0 (0.8 to 79.0)	4.0 (0 to 72.0)	9.9 (0.8 to 91.0)	4.9 (0.6 to 74.0)	nd
ERS, mm/h median (range)	11.0 (2.0 to 92.0)	14.0 (3.0 to 60.0)	18.0 (4.0 to 50.5)	20.0 (2.0 to 66.0)	nd
Immunosuppressant AZA:CYC:MTX:MMF	1:1:1:0	12:0:2:3	1:2:2:0	12:1:2:8	
Prednisolon, yes:no	7:32	11:28	13:25	13:25	
ANA, positive:negative	31:9		29:11		nd
ENA, positive:negative	10:30		10:30		nd
ACA, positive:negative	10:30		1:39		nd
ATA, positive:negative	3:32		7:29		nd
	5 unknown		4 unknown		
ARA, positive:negative	0:27		6:25		nd
	13 unknown		9 unknown		

lcSSc, limited cutaneous systemic sclerosis; dcSSc, diffuse cutaneous systemic sclerosis; ACA, anti to centromere antibody; COMP: cartilage oligomeric matrix protein; AU, arbitrary units; mRSS, modified Rodnan skin score; CRP, C-reactive protein; nd, not determined; ERS, erythrocyte sedimentation rate; AZA, azathioprine; CYC, cyclophosphamide; MTX, methotrexate; MMF, mycophenolate mofetil; ANA, antinuclear antibody; ENA, extractable nuclear antigen antibody; ATA, anti-topoisomerase antibody; ARA, anti-RNA polymerase III antibody.

Informed written consent was obtained from all participants involved in the study and permission was obtained from the regional ethical review board for Lund University. In the case of the patient under 18 years of age, oral consent was obtained from the parents for taking the first blood sample as part of clinical diagnostic routine as well as for storage of the sample for use in the research project, and informed written consent from the patient herself was obtained at the time point of the second sample when she was an adult. These procedures were fully in line with the ethics regulations in Sweden at the time of sampling.

### Fluorescence microscopy

Punch-biopsies, 3 or 4 mm in size were collected from the dorsal part of the right or left forearm, 2 to 4 cm proximal of the wrist. Skin samples were fixed in 4% formaldehyde, dehydrated with ethanol, embedded in paraffin and sliced to 4.5-μm sections. After mounting onto Superfrost Plus glass slides, samples were de-paraffinized and rehydrated as described [31]. Antigen retrieval was performed by heating samples to 100°C for 5 minutes in 0.01 M Na-citrate buffer, pH 6.0. Between each step of the staining, samples were washed three times with PBS and all antibodies were diluted in 1% BSA in PBS. After blocking sections with 1% BSA in PBS, sections were incubated for 1 h at room temperature (RT) with a monoclonal antibody against C3/C3b (ab11871, Abcam, Cambridge, United Kingdom), or an antigen affinity-purified rabbit anti-COMP antibody (homemade). Following washing, sections were incubated with Alexa Fluor-conjugated secondary antibodies (A21445, A1108, A21235, Invitrogen, Carlsbad, CA, USA) for 1 h at RT. Nuclear staining was performed by incubating samples with propidium iodide for 10 minutes at RT. Samples were analyzed and images obtained using a Zeiss LCM 510 confocal microscope. Co-localization of target molecules was evaluated using the CoLocalizer Express software (CoLocalization Research Software, Japan) and signal intensity in the tissues was measured using the ImageJ software.

### Measurement of serum COMP and COMP-C3b

Serum COMP-levels were measured using a commercially available COMP-kit (AnaMar, Lund, Sweden). Serum COMP-C3b was measured on maxisorp plates (Nunc, Thermo Scientific, Rockford, IL, USA) coated with a monoclonal antibody against COMP (home made) at a concentration of 5 μg/ml in 50 mM Hepes pH 7.4 with 2 mM CaCl_2_ overnight at +4°C. The plates were washed with 50 mM Tris–HCl, 150 mM NaCl, 0.1% Tween-20, pH 8.0 between each step in the assay. Plates were blocked using 1% BSA (Millipore, Billerica, MA, USA) diluted in 50 mM Hepes, pH 7.4 with 2 mM CaCl_2_ (blocking buffer) to prevent unspecific interactions. Serum samples were diluted 1:70 in 50 mM Hepes pH 7.4, 150 mM NaCl, 2 mM CaCl_2_, 2 mM MgCl_2_ with 50 μg/ml BSA, added to the wells and incubated for 2 h at RT. A biotinylated polyclonal anti-C3 antibody (CC7761, Sigma, St. Louis, MO, USA) was diluted in blocking buffer and incubated in the wells for 1 h at RT followed by a streptavidin-HRP conjugate (21130, Pierce, Thermo Scientific, Rockford, IL, USA). The plates were developed with *o*-phenylenediamine (OPD) substrate (Dako, Glostrup, Denmark) and H_2_O_2_ and the absorbance at 490 nm was measured using a Cary 50 MPR microplate reader (Varian, Palo Alto, CA, USA). Each sample was analyzed in duplicate and values obtained from the uncoated wells were subtracted from values obtained from antibody-coated wells. Obtained readings were then normalized against an internal control sample rendering data presented as arbitrary units (AU).

### Statistical analysis

The statistical significance of differences between groups was measured using the Kruskal-Wallis test with Dunn’s post hoc test or the Wilcoxon matched-pairs signed rank test, where appropriate. Two-parameter correlations were performed using Spearman’s correlation test. The significance of co-localization of staining in tissue was evaluated using Manders’ overlap coefficient.

## Results

### Levels of COMP-C3b complexes are elevated in SSc

**Figure 1:**
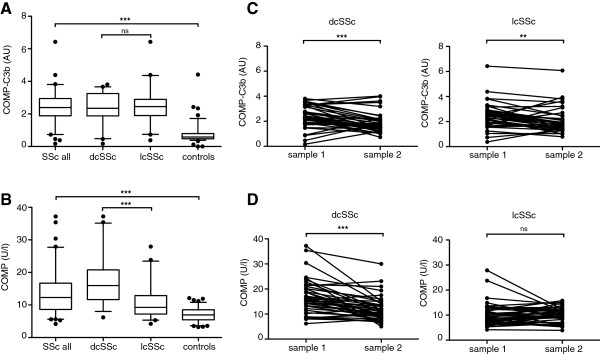
**Cartilage oligomeric matrix protein (COMP)-C3b and COMP in patients and controls.** Serum COMP-C3b **(A)** and COMP **(B)** were measured in patients with diffuse cutaneous systemic sclerosis (dcSSc) (n = 40), limited cutaneous systemic sclerosis (lcSSc) (n = 40) and healthy controls (n = 97). The longitudinal changes in COMP-C3b **(C)** and COMP **(D)** was measured. Statistical significance of differences was measured using the Kruskal-Wallis test with Dunn’s post hoc test **(A and B)** or the Wilcoxon matched-pairs signed rank test **(C and D)**. ns, not significant; ^**^P <0.01; ^***^, *P* <0.001.

Both COMP and COMP-C3b levels were found to be elevated in the serum of SSc patients compared to healthy controls (*P* <0.0001) corroborating our earlier results (Figure 1A and Table 1). There was no difference in serum COMP-C3b levels between patients with dcSSc and lcSSc, whereas patients with dcSSc had significantly higher serum COMP levels than patients with lcSSc (*P* <0.0001) (Figure 1B). COMP-C3b levels were not related to the autoantibody profile of the patient (not shown). There was a decrease in COMP-C3b in both dcSSc and lcSSc between the first and the second sample obtained (Figure 1C), however, this decrease was significant only for patients who were introduced to immunosuppressive treatment between the first and second sampling (dcSSc: *P* = 0.007 with immunosuppression, *P* = 0.188 with no immunosuppression; lcSSc: *P* = 0.0002 with immunosuppression, *P* = 0.077 with no immunosuppression). Prednisolone treatment did not affect the serum COMP-C3b levels in either patient group. Baseline COMP was high in the dcSSc-group and decreased significantly during follow up (Figure 1D). Patients with lcSSc, however, displayed normal COMP levels both at baseline and at follow up and consequently no significant reduction was observed.

### Levels of COMP-C3b complexes correlate with disease activity in dcSSc

**Figure 2:**
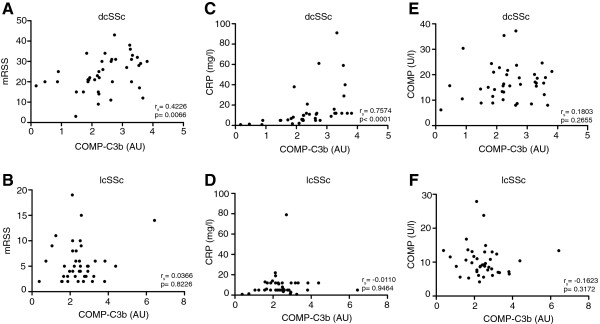
**Correlation of cartilage oligomeric matrix protein (COMP)-C3b with clinical parameters*.*** Correlation between serum COMP-C3b and modified Rodnan skin score (mRSS) **(A and B)**, CRP **(C and D)** and COMP **(E and F)** was measured in patients with disease duration less than 3 years. r_s_, Spearman’s correlation coefficient; dcSSc, diffuse cutaneous systemic sclerosis; lcSSc, limited cutaneous systemic sclerosis.

COMP-C3b correlated with mRSS in the dcSSc group at baseline (*r*_s_ = 0.4226, *P* = 0.0066) (Figure 2A) when a number of dcSSc patients had rather high mRSS values, but not at the later time point (Table 2). No correlation was found between these parameters in the lcSSc group (Figure 2B) or when studying all SSc-patients together (not shown). There was furthermore a correlation between COMP-C3b and CRP (*r*_s_ = 0.7574, *P* <0.0001) in the dcSSc group in the first time-point sample (Figure 2C). These data show that serum COMP-C3b, at least to some extent, is related to disease activity and inflammation, but mainly in the dcSSc subset of patients. The change in COMP-C3b between the first and second sample correlated weakly to the change in C-reactive protein (CRP) (*r*_s_ = 0.231, *P* = 0.043), but not to the change in mRSS (*r*_s_ = 0.007, *P* = 0.953). This indicates that COMP-C3b is more related to the general inflammatory response in the patients than to the actual skin fibrosis. The change in COMP, however, correlated to the change in mRSS (*r*_s_ = 0.362, *P* = 0.001) in agreement with earlier results [13].

**Table 2:** Correlation of COMP-C3b with clinical parameters

		**Spearman r**	***P*-value**
mRSS	all	0.1611	0.1534
	dcSSc	0.4226	0.0066
	lcSSc	0.0366	0.8226
CRP	all	0.3930	0.0003
	dcSSc	0.7574	<0.0001
	lcSSc	−0.0110	0.9464
COMP	all	0.0089	0.9374
	dcSSc	0.1803	0.2655
	lcSSc	−0.1623	0.3172
C3	all	0.2055	0.0749
	dcSSc	0.3145	0.0579
	lcSSc	0.0172	0.9171
C4	all	0.2184	0.0581
	dcSSc	0.3499	0.0338
	lcSSc	0.0935	0.5714

mRSS, modified Rodnan skin score; CRP, C-reactive protein; COMP, cartilage oligomeric matrix protein; dcSSc, diffuse cutaneous systemic sclerosis; lcSSc, limited cutaneous systemic sclerosis.

As we have observed earlier, COMP-C3b did not correlate with COMP, either in the whole SSc group or in any of the subgroups (Figure 2E-F). There was no correlation between COMP-C3b and the serum C3-levels in SSc patients, but a weak positive correlation was found between COMP-C3b and C4 (Table 2). Only five of the patients had C3 levels lower than 80% of normal at baseline, whereas 14 patients had C4-levels lower than 80% of normal, indicating that inflammation in SSc does not cause any major complement consumption.

### C3b is deposited in SSc skin lesions

**Figure 3:**
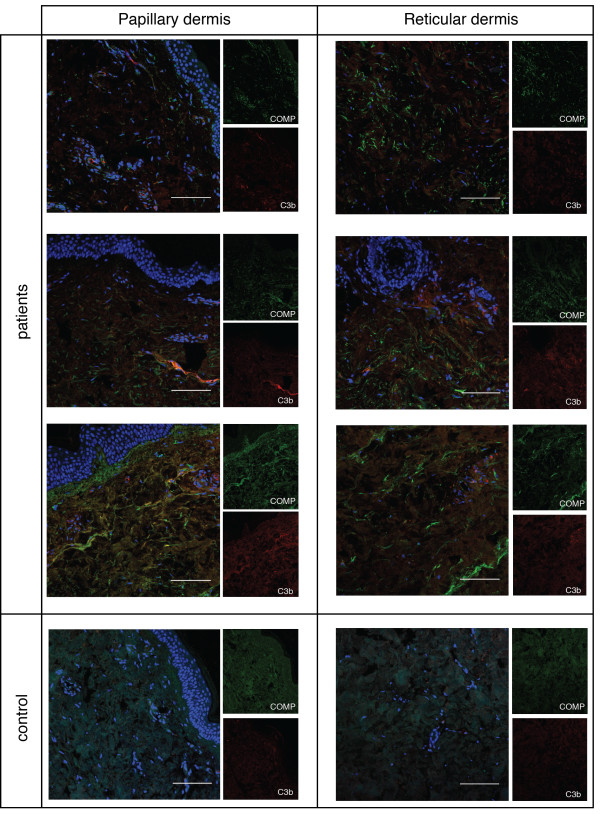
**Cartilage oligomeric matrix protein (COMP) does not co-localize with C3b in the skin*.*** Skin biopsies from seven patients with diffuse cutaneous systemic sclerosis (dcSSc) and three healthy controls were analyzed for COMP (green) and C3b (red). Nuclear staining is shown in blue. COMP staining was variable in the patients but mainly negative in the controls. Three patients and one control are shown as an example. Upper row, patient with dcSSc for 2 years, no current treatment; second row, patient with dSSc for 13 years, no current treatment; third row: patient with dcSSc for 2 years, one year of treatment with mycophenolate mofetil; fourth row, healthy control. The scale bars indicate 100 μm.

The expression of COMP in skin lesions of SSc patients has been demonstrated in several studies [4-6], where it was found both in papillary dermis as well as in deeper dermal layers. In healthy skin, COMP has been found mainly in the papillary dermis [8]. We evaluated COMP staining in skin biopsies from seven patients with dcSSc and three healthy controls. We found variable COMP staining throughout the tissue in patient samples. Two of the control samples were negative for COMP and the third showed a barely detectable staining (Figure 3).

C3b deposition was observed to a very low degree in patient samples and was in most cases more pronounced in the papillary dermis than in the reticular dermis (Figure 3). One of the three healthy controls also showed weak C3b staining, both in the papillary and reticular dermis. There was no statistically significant co-localization between COMP and C3b in the tissue, however, in two of the patients approximately 30% of the COMP and C3b stains overlapped. The amount of C3b deposition in the skin did not correlate with the local skin score.

As SSc is associated with autoantibody production, we evaluated whether autoantibodies deposit on COMP in the tissue. Similar to COMP, the IgG staining showed large heterogeneity between the patients and overlapped weakly with the COMP staining in only two samples (not shown).

## Discussion

We have confirmed in a large patient cohort that COMP-C3b levels are elevated in patients with SSc compared to healthy controls. The fact that there is no difference in serum COMP-C3b between patients with dcSSc and lcSSc is interesting, as these groups have quite different disease phenotypes and the systemic inflammatory component is more pronounced in dcSSc. The higher COMP levels seen in dcSSc are most likely a reflection of more extensive fibrotic skin involvement, as the COMP found in the circulation in SSc patients is thought to be released from the skin. Supporting this, we found a positive correlation between serum COMP and mRSS (*r*_s_ = 0.5808, *P* <0.0001). As we detect no correlation between COMP-C3b and COMP, we can hypothesize that only certain fragments of released COMP are able to activate complement or that a certain environment is required for the complexes to form. This is the case also in other diseases, such as rheumatoid arthritis, where no correlation was found between serum COMP and COMP-C3b [27]. We found a correlation between COMP-C3b and mRSS and CRP specifically in the dcSSc subset of patients but not in lcSSc patients. This indicates that COMP-C3b is more closely related to the disease activity and inflammation in patients with dcSSc. Interestingly, COMP-C3b still decreased longitudinally in both subsets of patients upon immunosuppressive treatment, showing that even though COMP-C3b is not directly correlated to individual parameters of inflammation, it may still act as a measure of disease activity, even in the lcSSc goup. As the change in COMP-C3b correlated only weakly to the change in CRP, we can hypothesize that even though both parameters measure inflammation, other as yet unknown factors affect the formation of COMP-C3b complexes that may not be detected by general inflammatory measures.

No correlation between serum COMP-C3b and C3 was found, most likely due to the fact that no apparent complement consumption was observed in the patients. In the dcSSc subset we saw a weak positive correlation between COMP-C3b and C4. Such positive correlation has previously been seen in patients with systemic lupus erythematosus [27]. Reasons for such a correlation remain speculative, but we can at least conclude that the formation of COMP-C3b complexes does not drive activation and therefore consumption of the classical complement pathway components.

Most skin biopsies of dcSSc patients studied showed COMP-staining, corroborating results published by others. Since we found very weak C3b staining in the skin of these patients, and the deposited C3b did not seem to co-localize with COMP, we can conclude that COMP in the skin does not drive measurable complement activation. Therefore, we can assume that the COMP-C3b complexes found in SSc patients do not originate from the skin but are formed in the blood after COMP is released into the circulation. It is likely that COMP needs to be cleaved or processed in a specific way or have a specific conformation for it to be able to trigger C3b-deposition, and that this does not occur until COMP is detached from the skin. These alterations might occur due to a specific inflammatory environment with local production of proteases and therefore may be down-regulated when the inflammatory reaction is diminished. This could explain the reduction of serum COMP-C3b in SSc patients at follow up. As the C-terminal globular domain of COMP, which is the domain responsible for complement activation [26], is engaged in multiple interactions in tissues, it is also possible that COMP needs to detach from its interacting partners in the tissue for it to be able to stimulate complement.

## Conclusion

Complexes between COMP and C3b are found in the circulation of both patients with dcSSc and lcSSc, although their levels relate more to individual inflammatory parameters in dcSSc. These complexes seem to form when COMP is released from the skin into the circulation and therefore it seems that COMP itself does not drive complement activation and deposition in the skin in SSc.

## Abbreviations

AU: Arbitrary units; BSA: Bovine serum albumin; COMP: Cartilage oligomeric matrix protein; CRP: C-reactive protein; dcSSc: Diffuse cutaneous systemic sclerosis; ELISA: Enzyme-linked immunosorbent assay; lcSSc: Limited cutaneous systemic sclerosis; mRSS: Modified Rodnan skin score; PBS: Phosphate-buffered saline; RT: Room temperature; SSc: Systemic sclerosis.

## Competing interests

The authors KEO, AMB, TS, and DH have filed a patent application on a method to detect tissue degradation leading to inflammation. Authors TS and DH are co-founders and own stocks in AnaMar.

## Authors’ contributions

KEO carried out the COMP-C3b ELISAs, stained and analyzed skin biopsies, did the statistical analysis, and drafted the manuscript. EH stained and analyzed skin biopsies, TS participated in the study design, and helped draft the manuscript. DH participated in the study design. RH participated in study design, provided patient material and revised the manuscript. AMB participated in the study design, helped draft the manuscript, and supervised the study. All authors read and approved the manuscript.

## Authors’ information

Kaisa E Otteby has previously published under the name Kaisa E Happonen.
